# The lateral habenula nucleus regulates pruritic sensation and emotion

**DOI:** 10.1186/s13041-023-01045-7

**Published:** 2023-06-27

**Authors:** Rui Chen, Xiang Xu, Xin-Yue Wang, Wen-Bin Jia, De-Shan Zhao, Na Liu, Zhen Pang, Xiao-Qing Liu, Yan Zhang

**Affiliations:** 1 Department of Neurology, The First Affiliated Hospital of USTC, Division of Life Sciences and Medicine, University of Science and Technology of China, Hefei, Anhui 230001 China; 2 School of Basic Medical Sciences, Division of Life Sciences and Medicine, University of Science and Technology of China, Hefei, Anhui 230027 China

**Keywords:** Lateral habenula nucleus, Glutamatergic neurons, Acute itch, Chronic itch

## Abstract

Itch is a complex aversive sensory and emotional experience. As a most upsetting symptom in many dermatological and systemic diseases, it lacks efficient treatments. The lateral habenula nucleus (LHb) encodes negative emotions in the epithalamus and has been implicated in pain and analgesia. Nevertheless, the role of the lateral habenula nucleus in the pruritic sensation and emotion remains elusive. Here we defined the crucial role of glutamatergic neurons within the lateral habenula nucleus (Glu^LHb^) in itch modulation in mice. We established histamine-dependent and histamine-independent models of acute pruritus, as well as the acetone-ether-water (AEW) model of chronic pruritus. We first assessed the effects of pruritogen injection on neural activation in both medial and lateral divisions of LHb in vitro. We then demonstrated that the population activity of Glu^LHb^ neurons was increased during the acute itch and chronic itch-induced scratching behaviors *in vivo.* In addition, electrophysiological data showed that the excitability of Glu^LHb^ neurons was enhanced by chronic itch. Chemogenetic suppression of Glu^LHb^ neurons disrupted both acute and chronic itch-evoked scratching behaviors. Furthermore, itch-induced conditioned place aversion (CPA) was abolished by Glu^LHb^ neuronal inhibition. Finally, we dissected the LHb upstream brain regions. Together, these findings reveal the involvement of LHb in processing both the sensational and emotional components of pruritus and may shed new insights into itch therapy.

**Supplementary Information:**

The online version contains supplementary material available at 10.1186/s13041-023-01045-7.

## Introduction

Itch (or pruritus) is defined as an intricate and aversive sensation of the skin, closely coupled with the scratch cycle. It comprises sensory, cognitive, emotional, and motivational components [1]. Pruritus is distinguished into acute and chronic itch [2]. Acute itch functions as an adaptive sensation to initiate appropriate motor reactions to avoid external danger or damage to the body. However, chronic itch, a most upsetting symptom in many dermatological and systemic diseases, provokes maladaptive suffering, such as severe skin lesions, sleep disruption, and even mental disorders [3–5]. Therefore, the itch dramatically impacts the quality of life. Although antihistamines are the first choice for anti-itch treatment, most chronic itch conditions resist antihistamines and remain a challenge clinically [6, 7]. Thus, further understanding of the mechanisms underpinning itch may aid in the development of effective therapies.

Itch and pain are two critical modalities of sensory input. Similar to pain, the itch information is transmitted from the peripheral afferents to the brain [8–11]. Extensive studies have pointed toward the mechanisms behind the sensory dimension of itch. The peripheral sensory fibers expressing TRPV1 (transient receptor potential vanilloid 1), spinal neurons expressing gastrin-releasing peptide receptor (GRPR), and a variety of cortical and subcortical areas including the parabrachial nucleus (PBN) [12], periaqueductal gray (PAG) [13], ventral basal nucleus of the thalamus (VB) [14], posterior thalamic nucleus (Po) [15, 16], medial prefrontal cortex (mPFC) [17, 18], and anterior cingulate cortex (ACC) [19], have been implicated in itch processing. However, the neural mechanisms underlying the emotional aspects of pruritus are less studied. In recent years, researchers unraveled the key role of emotion processing and regulating systems in itch-associated affection [13, 17, 20–24]. For example, a recent study reported that the GABA-containing and dopamine-containing neurons of the ventral tegmental area (VTA), an important reward center, are involved in encoding itch-associated aversion and itch relief-associated pleasure, respectively [22].

Despite these great progresses in exploring the central mechanisms of pruritus, the neural mechanisms underpinning the affective emotions of pruritus are still limited. The lateral habenula (LHb), also known as the “anti-reward center”, is a highly conserved nucleus across species that predominantly consists of glutamatergic neurons [25, 26]. It is commonly considered as a key integration hub for processing aversive information [27]. A functional MRI study showed that aversive itch stimulus can activate the lateral habenula [28]. The pivotal roles of LHb in pain processing and analgesia were also studied. For example, an aversive painful stimulus induces a strong Ca^2+^ transient in LHb neurons [29]. Under chronic pain conditions, the glutamatergic neurons in the LHb display hyperactivity [30, 31]. Furthermore, habenula is a relay in the descending pathway from the nucleus accumbens (NAc) to the PAG, subserving the analgesia via the opioidergic system [32]. Besides opioid analgesia, a recent study has demonstrated that intra-LHb injections of endogenous cannabinoid systemic agonists increase behavioral indicators [33]. These accumulating evidences suggest that LHb plays a crucial role in nociception. Given that the itch and pain are inextricably linked and the LHb activity is closely related to negative emotion, it is plausible to speculate that LHb may be involved in processing both the sensory and aversive itchy information.

In this study, we examined the population activity of LHb neurons using in vivo fiber photometry in responses to pruritogens and chronic itch in freely moving mice, and assessed the behavioral consequences following chemogenetic inhibition of Glu^LHb^ neurons in models of both acute and chronic itch. Furthermore, we explored the role of LHb in affective component of itch using the CPA paradigm. Using retrovirus tracing, we interrogated the upstream brain regions of LHb. Collectively, our results underscore that LHb is involved in processing both sensational and emotional components of acute and chronic itch.

## Results

### Acute pruriceptive stimulus activates Glu^LHb^ neurons

**Fig. 1:**
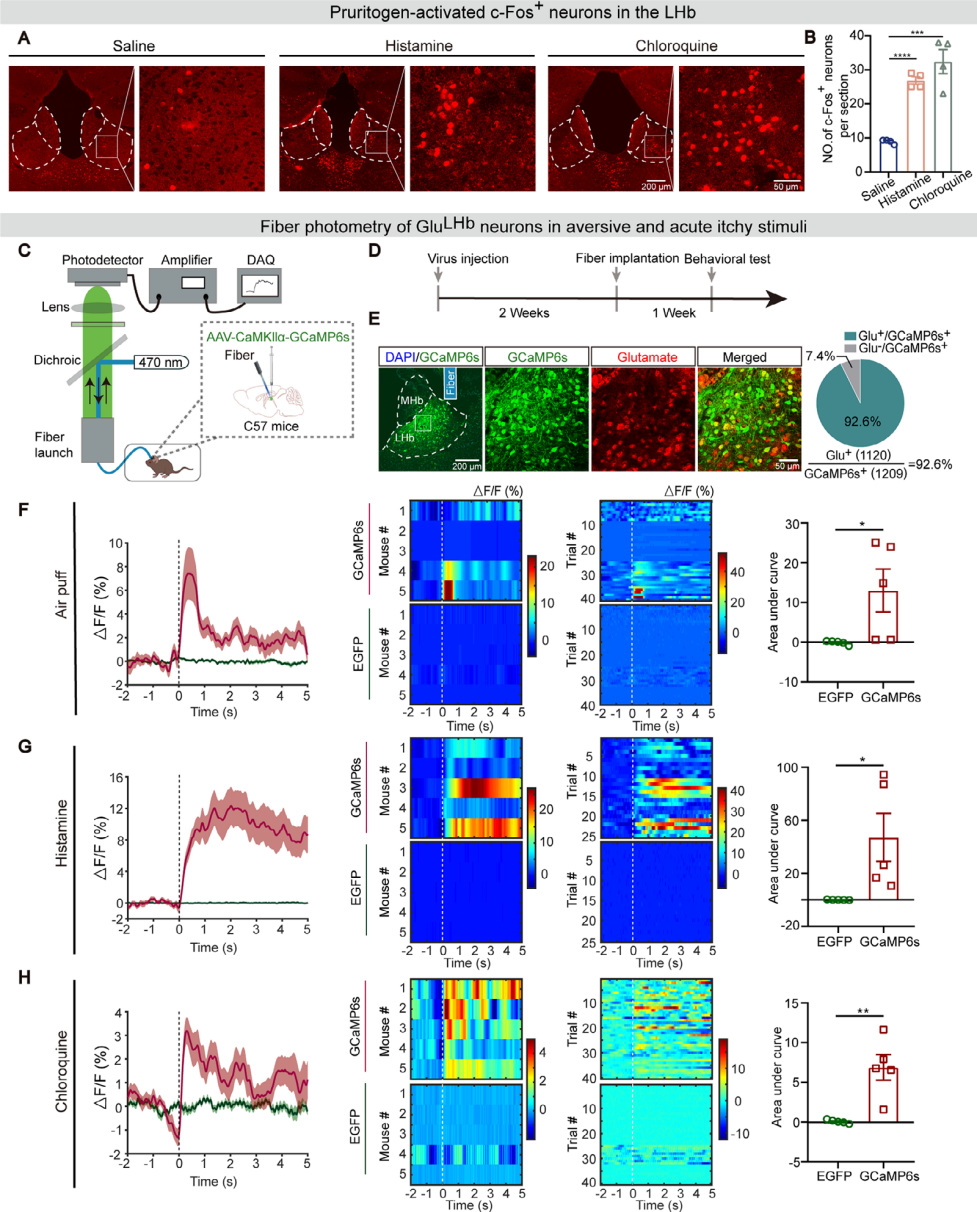
Acute pruriceptive stimulus activates Glu^LHb^ neurons. **A** Representative images of c-Fos immunostaining in pruritogen-treated or saline-treated mice. Scale bars, 200 μm and 50 μm. **B** Quantification of c-Fos staining in pruritogen-treated or saline-treated mice in the LHb (n = 4 mice per group). **C** Schematic depicting the fiber photometry apparatus, viral injection and optical fiber implantation for GCaMP6s expression used to measure Ca^2+^ signals in LHb CaMKIIα neurons. **D** Schematic diagram of the experimental design. **E** Left: representative immunofluorescence staining images showing viral expression and position of the fiber track in a C57 mouse. MHb: the medial habenula. Right: Quantification (pie chart) show double staining of anti-Glutamate and GCaMP6s in the LHb (n = 3 or 4 sections per animal from 4 mice). Scale bars, 200 μm (left) and 50 μm (right). **F** Left: Peri-event plots of the average change of Ca^2+^ fluorescence intensity when airing puff to the body. Vertical gray dashed line: onset of air puff stimulus; red, GCaMP6s virus channel recording; green, EGFP control virus channel recording; Middle: the heat map of Ca^2+^ transients aligned to the beginning of air puff in 40 trials from 5 mice (8 trials per mouse). Vertical white dashed line, onset of the air puff stimulus. Color scale indicates △F/F; Right: Quantification analysis of area under the curve (AUC) during 0–5 s of average Ca^2+^ fluorescence induced by air puff stimulus in the GCaMP6s group compared with the EGFP group. **G** The average Ca^2+^ fluorescence during histamine-evoked scratching. 25 trials from 5 mice were statistically analyzed (5 trials per mouse). Vertical gray and white dashed line: onset of the histamine-evoked scratching. **H** The average Ca^2+^ fluorescence during chloroquine-evoked scratching. 40 trials from 5 mice were statistically analyzed (8 trials per mouse). Vertical gray and white dashed lines: onset of chloroquine-evoked scratching. Significance was assessed by two-tailed unpaired Student’s *t*-test in (**B**, **F-H**), *p < 0.05, **p < 0.01, ***p < 0.001, ****p < 0.0001. Data were shown as mean ± SEM.

To analyze whether the LHb processes pruritic information, we first examined the response of the LHb neurons to pruritogens using c-Fos staining which is commonly used to assess neural activity. Chloroquine and histamine were used as pruritogens. The mice received intradermal injection of histamine (500 µg/50 µl), chloroquine (200 µg/50 µl) or saline (50 µl) into the rostral part of the back. c-Fos-positive neurons were then examined in the LHb. We demonstrated that substantial c-Fos-positive neurons were induced in the LHb by both pruritogens compared with vehicle-injected mice (Fig. 1A, B). The LHb consists of a medial (LHbM) and a lateral (LHbL) part [34]. Of note, histamine-induced c-Fos expression in the LHbM was significantly higher than that in the LHbL, while chloroquine-induced unbiased c-Fos expression in the LHbM and LHbL (Additional file: Fig. S1).

To further analyze whether the activated LHb neurons were behaviorally engaged in processing itchy information, we used fiber photometry with a video-tracking system to record the population activity of glutamatergic neurons in the LHb in response to pruritus. To achieve the goal, non-cre-dependent adeno-associated virus (AAV) expressing the calcium indicator GCaMP6s tethered to EGFP (AAV-CaMKIIα [calmodulin-dependent protein kinase II]-GCaMP6s-EGFP) or AAV-CaMKIIα-EGFP was injected into the unilateral LHb of C57BL/6J mice, and optical fibers were implanted above the LHb, enabling long-term recordings of intracellular Ca^2+^ transients (Fig. 1C, D). *Post hoc* staining showed that ~ 93% of GCaMP6s-positive neurons overlapped with Glu^LHb^ neurons (Fig. 1E), demonstrating the high specificity of viral targeting.

As previously described [35], air puff (an aversive stimulus) induced remarkable increases in Ca^2+^ transients of Glu^LHb^ neurons in the GCaMP6s group compared with EGFP group (Fig. 1F), suggesting the participation of Glu^LHb^ neurons in encoding aversive emotions. To assess the potential role of these neurons in acute pruritus-induced scratching trains, histamine (500 µg/50 µl) was injected into the anterior region of the back and the mice displayed robust scratching behaviors by a hind paw (Additional file: Fig. S2A). By aligning the fluorescent calcium signals with the video footage of scratching onset, we found that intracellular Ca^2+^ in Glu^LHb^ neurons was significantly elevated at approximately the same time as the mice started scratching, and the Ca^2+^ elevation sustained for a few seconds. However, we did not observe any changes in the Ca^2+^ transients of Glu^LHb^ neurons in EGFP-expressing control mice (Fig. 1G). To further confirm the involvement of Glu^LHb^ neurons in itch processing, we tested the responsiveness of Glu^LHb^ neurons to non-histamine-dependent pruritus (Additional file: Fig. S2B). As expected, the Glu^LHb^ neurons obtained the similar pattern of responses as histamine following intradermal injection of chloroquine (200 µg/50 µl) into the nape (Fig. 1H). Together, these results indicated that neuronal activity of Glu^LHb^ neurons is associated with scratching behaviors during acute itch.

### Chronic itch enhances Glu^LHb^ neural excitability

**Fig. 2:**
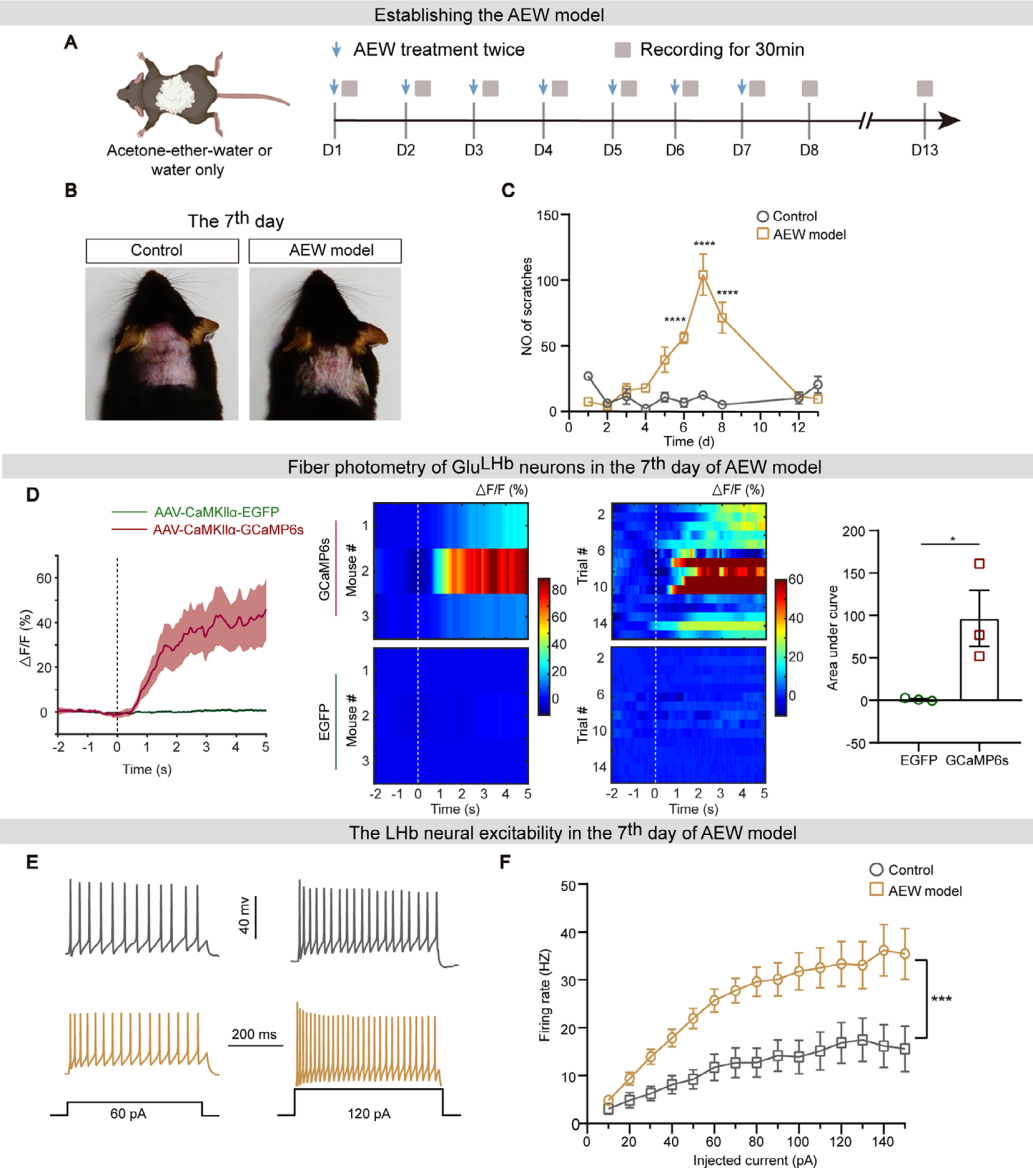
Chronic itch enhances Glu^LHb^ neural excitability. **A** Schematic diagram of the experimental design. **B** Representative images of dorsal neck skin of the water only (Control) and acetone-ether-water (AEW)-treated mice on the seventh day. **C** Timeline of scratch bouts in the control and AEW groups in C57 mice. **D** Left: Peri-event plots of the average change of Ca^2+^ fluorescence intensity in the AEW model. Vertical gray dashed line: onset of AEW-evoked scratching; red, GCaMP6s virus channel recording; green, EGFP control virus channel recording; Middle: the heat map of Ca^2+^ transients aligned to the beginning of scratching in 15 trials from 3 mice (5 trials per mouse). Vertical white dashed line, onset of AEW-evoked scratching. Color scale indicates △F/F; Right: Quantification analysis of area under the curve (AUC) during 0–5 s of average Ca^2+^ fluorescence induced by AEW-evoked scratching stimulus in the GCaMP6s group compared with the EGFP group. **E, F** Representative a trace (**E**) and summary data for the firing rates (**F**) of Glu^LHb^ neurons recorded from AEW or control mice. (Control, n = 21 cells; AEW, n = 24 cells). *p < 0.05, ***p < 0.001, ****p < 0.0001, not significant (ns). Two-way ANOVA with Bonferroni’s correction for multiple comparisons in (**C, F**) and two-tailed unpaired Student’s *t*-test in (**D)**. Data were shown as mean ± SEM

An acetone-ether-water (AEW) model is commonly used as a chronic pruritus model [36]. We first applied an acetone/ether mixture topically, followed by water to establish the AEW pruritus model. With the extension of the modeling time, the moisture on the skin surface was gradually decreased and the dryness was most severe on the seventh day of the model (Fig. 2A, B). Behaviorally, significant bouts of scratching were observed on the sixth day of AEW treatment compared with control mice, and the pruritic behaviors peaked on the seventh day and disappeared on the twelfth day after the initial AEW treatment (Fig. 2C).

To investigate the role of Glu^LHb^ neurons in encoding chronic itch, we assessed the calcium activity of Glu^LHb^ neurons after establishing AEW model. Following seven days of the AEW modeling, mice developed itchy behaviors (Additional file: Fig. S2C), and Glu^LHb^ neurons displayed a significantly calcium dynamics aligned to the onset of scratching (Fig. 2D), implying that Glu^LHb^ neural activity is also associated with scratching behaviors during chronic itch.

Next, we assessed Glu^LHb^ neuronal excitability by whole-cell recordings performed in slices from AEW-treated or control mice. Compared with controls, the firing rate of Glu^LHb^ neurons were remarkably increased in slices obtained from AEW-treated mice (Fig. 2E, F), suggesting a potential role of Glu^LHb^ neural hyperactivity in pathophysiology of AEW-induced chronic itch.

### Chemogenetic inhibition of Glu^LHb^ neurons suppresses pruritogens and chronic pruritus-induced scratching behaviors

**Fig. 3:**
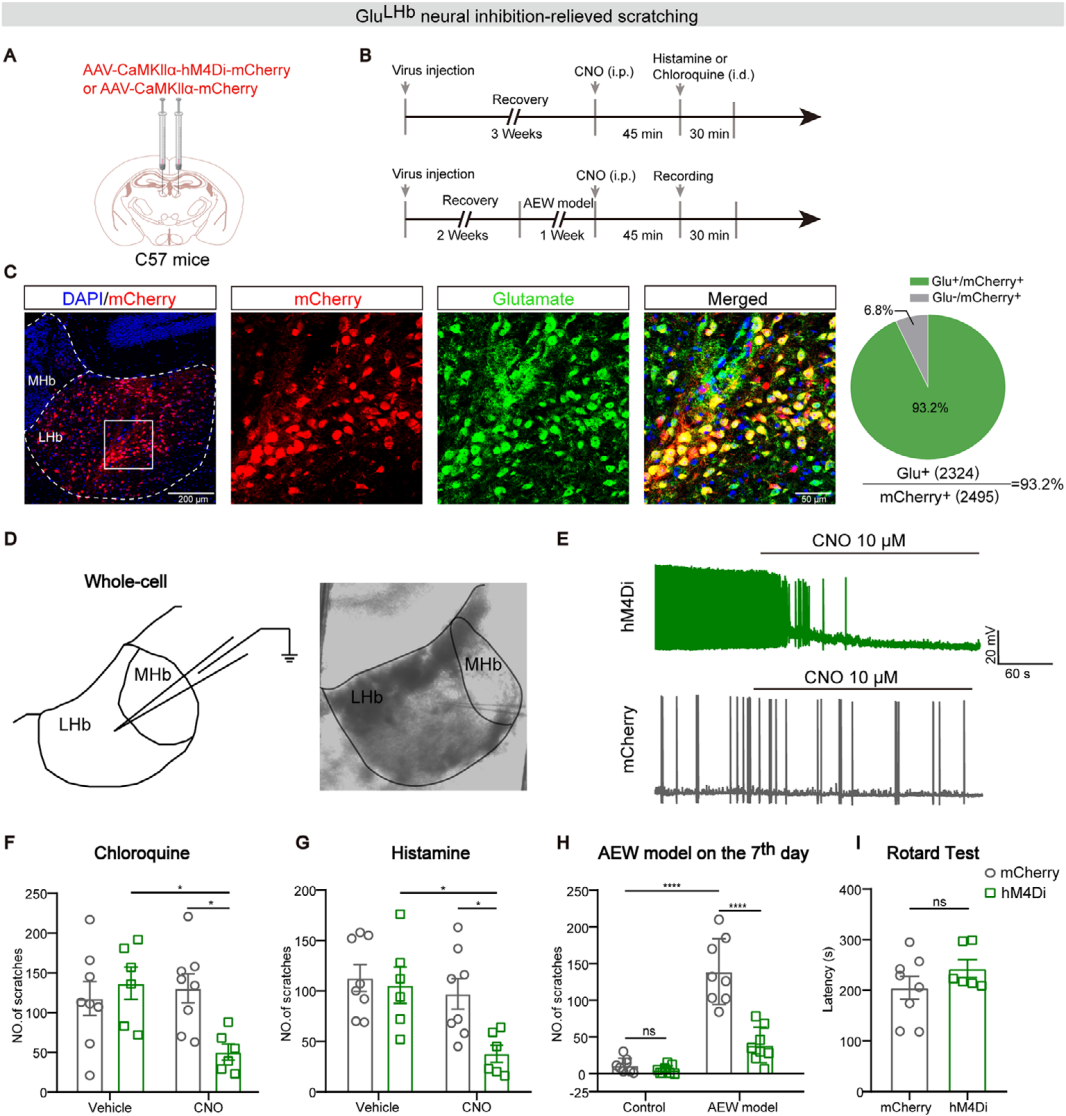
Chemogenetic inhibition of Glu^LHb^ neurons suppresses pruritogens and chronic pruritus-induced scratching behavior. **A** Schematic diagram of bilateral injection of AAV-CaMKIIα-hM4Di-mCherry or AAV-CaMKIIα-mCherry into the LHb. **B** Schematic diagram of the experimental design. **C** Left: Representative histological images and summary data for the percentage of mCherry^+^ neurons which co-localize with anti-Glutamate in the LHb. (n = 3 sections per animal from 4 mice). Scale bars, 200 μm and 50 μm. **D** Schematic showing whole-cell recording of hM4Di-mCherry^+^ cells in acute brain slices. **E** Effect of bath application of 10 µM CNO on the membrane potential and action potentials of a hM4Di^+^ neuron (top) or a mCherry^+^ neuron (bottom) in the LHb. **F, G** Chemogenetic inhibition of Glu^LHb^ neurons significantly impaired scratching induced by intradermal injection of histamine (**F**) and chloroquine (**G**) into the nape. (hM4Di group, n = 6 mice; mCherry group, n = 8 mice). **H** Effects of chemogenetic inhibition of Glu^LHb^ neurons on spontaneous scratching behavior produced by AEW treatment on the 7th day. **I** Chemogenetic manipulation of Glu^LHb^ neurons had no effect on the motor ability in rotarod test. Significance was assessed by two-way ANOVA with Bonferroni’s multiple comparisons test in (**F-H**) and Mann-Whitney U test in (**I**), *p < 0.05, ****p < 0.0001, not significant (ns). All data were shown as mean ± SEM

To further investigate the function of Glu^LHb^ neurons in processing itch, we applied chemogentic method to modulate Glu^LHb^ neurons after the pruritogen injection or AEW modeling. To suppress the neuronal activities of LHb, we used an inhibitory hM4Di system. The hM4Di-mCherry, delivered by AAVs, was expressed under CaMKII promoter based on that the major cell type of LHb is glutamatergic (Fig. 3A, B). Statistics summary showed that the high overlapping of neurons expressing hM4Di-mCherry with anti-Glutamate staining, demonstrating high viral specificity (Fig. 3C). To clarify the efficiency of an inhibitory hM4Di system, whole-cell recordings were performed from LHb containing brain slices and we found that bath application of clozapine-N-oxide (CNO, 10 µM) hyperpolarized the membrane potential of the hM4Di^+^ neurons while the mCherry^+^ neuron was not affected in the control groups (Fig. 3D, E).

We next examined the influence of Glu^LHb^ neuronal inhibition on itch-induced scratching behaviors. We recorded the scratching behaviors evoked by intradermal injection of pruritogens (histamine and chloroquine) into the nape of the neck 45 min after intraperitoneal (i.p.) injection of CNO (2 mg/kg) or saline. Compared with the vehicle injection group, pretreatment of CNO significantly reduced scratching responses evoked by both the chloroquine and histamine in hM4Di group. However, mCherry controls did not display any changes in scratching events (Fig. 3F, G). These data suggested that the Glu^LHb^ neurons were involved in pruritogens-induced scratching behaviors.

We next sought to examine whether LHb is also required for the itchy response induced by the AEW model. Thus, AAV-CaMKIIα-hM4Di-mCherry or AAV-CaMKIIα-mCherry was bilaterally injected into the LHb (Fig. 3A, B). After seven days of continuous treatment with AEW, CNO (2 mg/kg) was intraperitoneally injected to suppress neuronal activities expressing hM4Di (Fig. 3B). We found that that inhibition of LHb activity significantly alleviated AEW-induced scratching bouts. For controls, further intraperitoneal injection of CNO did not affect scratching behavior (Fig. 3H). Moreover, chemogenetic inhibition of Glu^LHb^ neurons did not alter the locomotor ability of mice using the rotarod test (Fig. 3I). Thus, Glu^LHb^ neurons were also involved in chronic itch behaviors.

### Glu^LHb^ neural inhibition blocks the itch-associated aversiveness

**Fig. 4:**
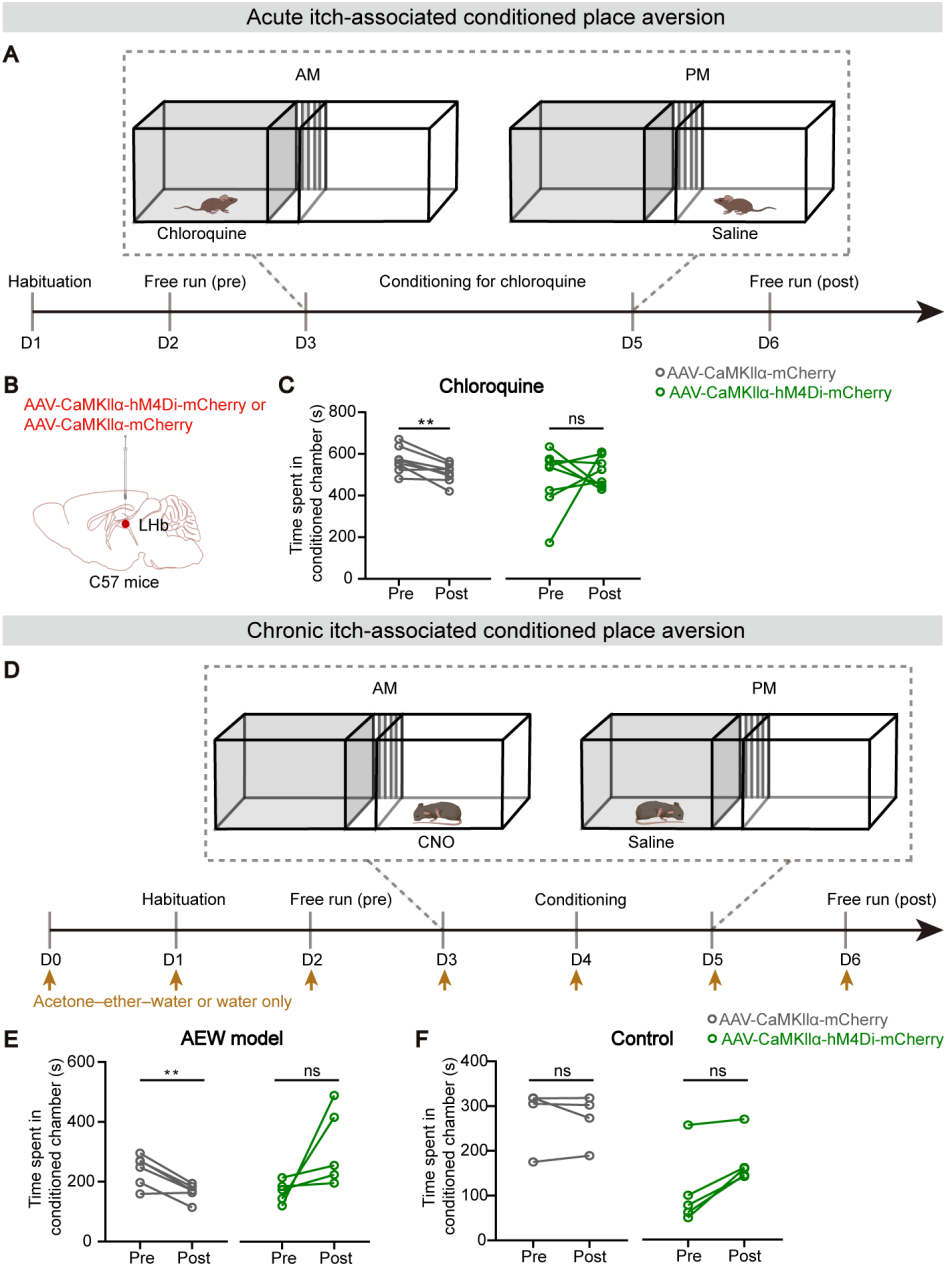
Glu^LHb^ neural inhibition blocks the itch-associated aversiveness. **A** An outline of the experimental procedure for chloroquine-induced conditioned place aversion (CPA) test. **B** Viral targeting of AAV-CaMKIIα-hM4Di-mCherry or AAV-CaMKIIα-mCherry bilaterally into the LHb of C57 mice. **C** Effect of Glu^LHb^ neural inhibition on the acute itch-associated CPA. (hM4Di group, n = 8 mice; mCherry group, n = 8 mice). **D** An outline of the experimental procedure for chronic itch-induced CPA test. **E** Effect of Glu^LHb^ neural inhibition on the AEW group-associated CPA. (hM4Di group, n = 5 mice; mCherry group, n = 5 mice). **F** Effect of Glu^LHb^ neural inhibition on the AEW control group-associated CPA. (hM4Di group, n = 5 mice; mCherry group, n = 5 mice). Significance was assessed by two-tailed paired Student’s *t*-test in (**C, E**) and Wilcoxon signed-rank test for paired test in (**F**), **p < 0.01, not significant (ns). All data were shown as mean ± SEM

The sensation of itch consists of both sensory and emotional components. It has been shown that itch can induce aversion in mice which requires peripheral itch signal inputs [13, 23]. However, whether LHb is required for itch-associated affective responses remains unknown. To examine the role of Glu^LHb^ neurons in the negative reinforcement of acute itch, we performed a conditioned place aversion (CPA) paradigm (Fig. 4A). One chamber was defined as itch-unpaired (injection of saline) and the other as itch-paired (injection of chloroquine). After free access to both chambers on day 1, mice were conditioned for 3 days, by administering either saline or chloroquine. On conditioning days, mice were pre-treated with either saline or CNO (i.p) for 45 min before being paired in the specified chambers. We expressed mCherry^+^ (control) or hM4Di^+^ in Glu^LHb^ neurons in C57 mice (Fig. 4B). After conditioning sessions, control mice spent significantly less time in the itch-paired chamber during the post-training than pre-training test, demonstrating the successful establishment of acute pruritus-associated aversive response. Interestingly, chemogenetic inhibition of Glu^LHb^ neurons effectively blocked the acute itch-associated CPA (Fig. 4C).

We next asked whether inhibition of Glu^LHb^ neurons would also abolish place aversion to chronic pruritus in AEW model. In the process of the AEW model treatment (0–6 days), we used an identical CPA procedure (Fig. 4D). Briefly, mice were placed in a 3-chamber CPA arena and given free access to entire chambers for 30 min on day one. The apparatus was re-designated as a CNO-paired chamber in which the mice were injected with CNO (i.p) and the other as saline-paired chamber. From day 3 to day 5, mice were conditioned for 3 continuous days with two pairing sessions each day. 24 h after the last pairing, mice were given free access to all chambers, and the time spent in the CNO-paired chamber was analyzed. Animals expressing mCherry spent significantly less time in the CNO-paired chamber in which they were treated with AEW, suggesting the establishment of chronic itch-associated CPA. Intriguingly, we found that animals expressing the stimulatory hM4Di in the Glu^LHb^ neurons spent no difference time in the CNO-paired chamber, indicating the abolishment of the established place aversive (Fig. 4E). To confirm that abolishment of chronic itch-associated CPA is resulting from itch relief by LHb neural inhibition, but not attributed to the simple offset by LHb neural inhibition-induced reward, we performed the similar conditioning procedure in the AEW control group (water-treated mice) (Fig. 4D). We demonstrated that LHb neural inhibition did not evoke any preference or aversion in control mice (Fig. 4F).

Collectively, Glu^LHb^ neurons are involved in processing both the sensory and affective dimensions of acute and chronic pruritus.

### LHb upstream brain areas

Based on the fact that LHb plays a key role in processing and regulating itch information above, we finally investigate how the itchy signals are transmitted to the LHb. In order to dissect the LHb upstream brain areas, we injected AAV2/2-retro-hSyn-EGFP into the LHb of C57 mice. Three weeks following injection of the retrograde virus, LHb-projecting neurons were labeled. Animals were decapitated and an unbiased whole-brain screening of EGFP signals was performed (Additional file 3: Fig. S3A). We found that LHb received broad presynaptic inputs from various brain regions, mainly including the anterior cingulate cortex (ACC), medial prefrontal cortex (mPFC), ventral tegmental area (VTA), entopeduncular nucleus (EPN) and lateral hypothalamus (LHA) (Additional file 3: Fig. S3C). After quantitatively analyzing the distribution of the upstream brain region, we found that EGFP positive neurons were mainly located in ipsilateral sites (Additional file 3: Fig. S3B). These data indicate that they are most likely candidates processing itchy information to LHb.

## Discussion

Although brain imaging techniques have shown that LHb is activated by pruritogen [28], there is no direct evidence that LHb is involved in processing pruritic information. In our study, we revealed a previously unknown role of LHb in itch regulation through three major findings. First, Glu^LHb^ neurons are activated by pruritogens, and the excitability of Glu^LHb^ neurons is enhanced by chronic itch. Second, Glu^LHb^ neural activity is required for full expression of both acute and chronic pruritus. Third, Glu^LHb^ neural activity is necessary for aversive itch component assessed by CPA paradigm.

LHb was anatomically divided into medial and lateral parts in early 1977 [34]. Their afferents and efferents are topographically organized [26, 37]. In Ko’s study, histamine injection preferentially activates neurons located in the restricted regions of medial LHb. In comparison, we observed even distribution of c-Fos^+^ neurons in the medial and lateral LHb in chloroquine-induced pruritus (Additional file 1: Fig. S1). It’s possible that distinct population of LHb neurons and their respective upstream and downstream brain regions are involved in processing different types of pruritogens. Glu^LHb^ neurons are also functionally heterogeneous with respect to their response to aversive stimuli via single-unit recording and fiber photometry [29]. As previous experiments, puffing the air to the body part increased the calcium activity of LHb (Fig. 1F), indicating that LHb might be involved in encoding aversive emotion as well. Pruritic irritations also immediately elicited an activation of Glu^LHb^ neurons closely associated with the beginning of a scratching bout. Interestingly, the amplitude of the calcium signals is very different between histamine vs. chloroquine. In common with that histamine-independent pruritus emerges the more aversive sensation [23], histamine-induced itch elicited stronger activation of Glu^LHb^ neurons compared to chloroquine-induced itch (Fig. 1G, H). We proposed that different subpopulations of LHb neurons are involved in processing histamine-dependent and histamine-independent itch. Moreover, the increased Ca^2+^ signal lasted for a few seconds. We speculated that the unpleasant or aversive aspect of itch that initiates scratching may cause the sustained Ca^2+^ signals. However, a drawback of the fiber photometry method is that the recorded signals were derived from multiple neurons in LHb, which might lead to missing or masking any heterogeneity in the responses of individual neurons. It will be exciting for future studies to explore the identity of histamine/chloroquine-specific and sensory/emotional itch-specific neurons using miniscope, multi-channel extracellular recordings, microendoscope and activity-dependent cellular labeling method.

Chemical itch can be classified into two subtypes, histaminergic and nonhistaminergic [2]. To investigate the potential different roles of LHb in histaminergic and nonhistaminergic itch processing, we thus used histamine as a histaminergic pruritogen and chloroquine as a nonhistaminergic pruritogen. All of these pruritogens have been widely used in previous studies [38], and the chosen concentration could steadily induce itch-related scratching without causing pain-related wiping or skin lesions in mice. Cell-type-specific inhibition of LHb neurons decreased the scratching behavior in both histamine- and chloroquine-induced acute itch (Fig. 3F, G). Rotarod tests were also performed to confirm that the reduction in scratching behavior was not caused by a motor defect (Fig. 3I). The behavioral result of our chemogenetic experiments is consistent with the increased population activity by chemical pruritogens. Furthermore, we showed that itch evoked by chloroquine administration and AEW model resulted in robust conditioned place aversion (Fig. 4C, E), demonstrating that itch is fundamentally an aversive sensation. Importantly, chemogenetic inhibition of Glu^LHb^ neurons relieves the aversive component of itch experience through validation of the itch-induced CPA paradigm.

It is worthy of mention that extensive distribution of LHb upstream neurons was observed in ACC and mPFC using the retrograde labeling strategy (Additional file 3: Fig. S3B, C), which were previously shown activated by itch and involved in itch regulation via descending pathway to the spinal cord [39–41]. It is possible that the LHb functionates as a relay center for itch information transmitting from the ACC and mPFC. Moreover, we found that the EPN, LHA and VTA also innervate the LHb. The EPN is the primary source of the negative signals [42, 43]. The LHA is targeted by spinothalamic tract [44], and is implicated in itch information processing [45]. VTA GABA neurons regulated scratching behaviors by encoding pruritus-related aversion [22]. From above, the LHb receives direct input from nuclei associated with pruritus. Apart from the direct pathway, more complex wiring involved in itch transmission could also occur. A recent study has shown that the VTA and LHb play critical roles in the transmission of the reward and aversion as the downstream target of the LHA in divergent efferent pathways [46]. Thus, the VTA-LHb, as well as the LHA-VTA-LHb paralleled neuronal circuits might be involved in signaling itch information. Nevertheless, manipulation of the-LHb centered circuits will be required to determine their roles in itch in the future.

Relative to acute itch, pathophysiological basis underlying chronic itch has not been well determined, therefore it is an essential step in addressing the clinical burden of chronic itch [3]. We demonstrated similar results in a model of chronic itch, where inhibition of LHb glutamatergic neurons resulted in attenuation of spontaneous scratching behavior produced by AEW model and abolishment of the itch-associated aversion (Fig. 3H, and 4E, F). Moreover, we demonstrated the enhanced excitability of LHb glutamatergic neurons in chronic itch (Fig. 2E, F). Since LHb neurons are very heterogenous, it is interesting to define the distribution of the hyperactive LHb neurons in chronic itch in the future. These findings potentially provide a new therapeutic target for chronic itch. Exploring the efficacy of LHb manipulation on the chronic itch in humans are anticipated.

Taken together, our current observations identify a critical role of the LHb in itch modulation wherein selective inhibition of LHb glutamatergic neurons produces itch relief, which allow us to better understand the central neural circuit mechanism of the itch sensation and potentially aids to develop effective treatment for pathological itch such as atopic dermatitis.

## Materials and methods

### Experimental animals

All surgical and experimental procedures were approved by the Animal Care and Use Committee of the University of Science and Technology of China. In this study, wild-type 6–10 weeks old male C57BL/6J mice (purchased from Beijing Vital River Laboratory Animal Technology) were used. Animals were maintained under a 12-h light-dark cycle (lights on from 7:00 to 19:00) with standard lab mouse pellet food and water available *ad libitum*. Littermate mice were split into random groups before all behavioral experiments. After experiments, animals were euthanized by an extra dose of pentobarbital (2.5%; i.p) or isoflurane (5%; inhaling). All efforts were made to minimize the number and suffering of experimental animals.

### Drug preparation and administration

Chloroquine and histamine were purchased from Sigma-Aldrich (St. Louis, MO) and dissolved in sterile saline solution. The clozapine-N-oxide (CNO) was purchased from APExBIO Technology LLC (Houston, Texas) and dissolved in saline after gently mixing with a vortex. Other detailed information for time and doses for their use was indicated in results or figure legends.

### Animal models

#### Acetone-ether-water model (AEW)

The acetone-ether-water model is commonly used as a non-histamine-dependent pruritus model to study chronic pruritus. We treated the mice with AEW model as previously reported [36, 47]. The hair of mice was shaved over the rostral part of the back until the skin was completely exposed at least 3 days before the experiment. Cotton (2 × 2 cm) soaked with a mixture of acetone and diethylether (1:1) was applied to the shaved area for 15 s, followed immediately after AE treatment, cotton soaked with distilled water was laid upon the same area for 30 s (AEW group). For the control group, only cotton soaked in water was used for 45 s instead. The animals were treated twice daily (9:00 and 17:00). Mice with chronic dermatitis show spontaneous scratching all day long. Thus, we observed scratching of the mouse at least 14 h after the treatment for barrier disruption on the previous day.

#### Neck models of acute itch

Neck models of acute itch behavioral tests were recorded and analyzed as previous studies [22, 48]. In brief, mice were shaved on the scruff of the neck before testing. Four mice were put individually into an acrylic box composed of four cells the chamber and allowed to habituate for 30 min. Then, the mice were briefly removed from the chamber and intradermally injected with the desired pruritic agent. Scratching was recorded with a digital video camera for 30 min. The video was then played back for blindly manual analysis and quantified the number of scratching bouts. A scratching bout represents a lifting of hindquarter to rub the injected site of the body and then placing it on the floor.

#### Stereotaxic surgery

All stereotaxic injections were performed using a stereotaxic frame (RWD, Shenzhen, China) which mice were deeply anesthetized with with 3% pentobarbital sodium (30 mg/kg, i.p.) were mounted on. A heating pad was used to maintain the core body temperature of the animals at 36 °C. Ophthalmic ointment was applied to maintain eye lubrication throughout the surgery. We used the dental drill to carefully remove the skull above the LHb region. A volume of 100 nl viruses (depending on the expression strength and viral titer) were injected at a rate of 50 nl/min using a micro-infusion pump (micro, WPI) by connecting to calibrated glass microelectrodes (tip diameter of 10–20 μm). After the injection, the glass pipettes were left in place for 5–10 min before withdrawal to allow for diffusion. The animals were allowed to recover from anesthesia on a heating blanket before returning to their home cage. Dorso-ventral (DV) from the brain surface, anterior-posterior (AP) from bregma and medio-lateral (ML) from the midline (in mm) were the coordinates.

#### Viral injection and optical fibers implantation

For chemogenetic inhibition of LHb glutamatergic neurons, mice were bilaterally microinjected with 120 nl rAAV2/9-CaMKIIα-hM4Di-mCherry-WPREs (titer: 5.91E + 12 vg/ml, BrainVTA, PT-0050), and rAAV2/9-CaMKIIα-mCherry-WPRE-pA (titer: 5.14E + 12 vg/ml, BrainVTA, PT-0108) as a control into the LHb. And then mice were injected with CNO (2 mg/kg, i.p.) to manipulate the activity of the LHb. To retrograde LHb-projecting neurons, the unilateral LHb of C57BL/6J mice received microinjection of 120 nl AAV2/2-retro-hSyn-EGFP-WPRE-pA (titer: 5.18E + 12 vg/ml, BrainVTA, PT-1990).

To record calcium fluorescence of LHb glutamatergic neurons, the unilateral LHb (AP: -1.9 mm, ML: -0.45 mm, DV: -2.65 mm) of C57BL/6J mice received microinjection of 120 nl rAAV2/9-CaMKIIα-GCaMP6s-WPRE-pA (titer: 5.31E + 12 vg/ml, BrainVTA, PT-0110) or control virus rAAV2/9-CaMKIIα-EGFP-WPRE-pA (titer: 5.82E + 12 vg/ml, BrainVTA, PT-0959). After 14 days of virus injection, an optic fiber (diameter: 200 μm; length, 4 mm; N.A., 0.37; Inper) was implanted 100 μm above the viral injection site. The optical fiber was secured with skull-penetrating screws and dental acrylic. Photometric recordings were conducted using the fiber photometry recording system after the fibers-implantation procedures to ensure adequate animal recovery.

### *In vitro* electrophysiology

#### Slice preparation

Animals were anesthetized using the intraperitoneal administration of pentobarbital sodium (30 mg/kg) followed by intracardially perfused with ~ 20 ml of ice-cold oxygenated Nmethyl-d-glucamine (NMDG)-based artificial cerebrospinal fluid (ACSF) composed of the following (in mM): 93 NMDG, 1.2 NaH_2_PO_4_, 2.5 KCl, 20 HEPES, 30 NaHCO_3_, 2 thiourea, 25 glucose, 3 Na-pyruvate, 5 Na-ascorbate, 10 MgSO_4_, 0.5 CaCl_2_, and 3 glutathione (GSH). The 300-µm-thick coronal brain slices containing the LHb were cut in ice-cold cutting solution sectioned at 0.34 mm/s using a Leica VT1000s vibratome. Slices were initially warmed in cutting solution at 33 °C for 10 min and then placed into N-2-hydroxyethylpiperazine-N-2-ethanesulfonic acid (HEPES) ACSF that contained (in mM) 92 NaCl, 2.5 KCl, 30 NaHCO_3_, 20 HEPES, 1.2 NaH_2_PO_4_, 25 glucose, 2 MgSO_4_, 2 CaCl_2_, 2 thiourea, 3 Na-pyruvate, 5 Na-ascorbate, and 3 GSH at 25 °C for at least 1 h. After incubation, the slices were transferred into an immersion recording chamber (Warner Instruments, USA) and were continuously perfused with normal ACSF that contained (in mM) 2.4 CaCl_2_, 3 KCl, 129 NaCl, 1.3 MgSO_4_, 1.2 KH_2_PO_4_, 20 NaHCO_3_and 10 glucose at a rate of 2.5-3 ml/min at room temperature. During preparation and recording, all solutions were continuously bubbled with 95% O_2_/5% CO_2_ to maintain stable hydrogen and continuously provide oxygenation.

#### *In vitro* electrophysiological recordings

Whole-cell patch-clamp recordings on the target LHb neurons were visually guided by IR-DIC visualization and an infrared-sensitive charge-coupled device (CCD) camera using a fluorescent Olympus BX51WI microscope. Recording pipettes (5–8 MΩ) were pulled from glass capillaries using a four-stage horizontal micropipette puller (P1000, Sutter Instruments) and backfilled with intracellular solution containing (in mM) 130 K-gluconate,10 HEPES, 5 KCl, 0.6 EGTA, 2 MgCl_2_, 2 Mg-ATP, and 0.3 Na-GTP. Recordings were acquired with a Multiclamp 700B amplifier (Molecular Devices). Data were sampled at low-pass filtered at 2 kHz, digitized at 10 kHz. Further analysis was performed offline by Clampfit 10.0 software (Molecular Devices). These recordings were excluded when more than 20% of changes in the series resistance occur. CNO (10 µM) was bath-applied to confirm the efficacy of hM4Di-mediated inhibition. To record firing rates of neuronal excitability, 10 to 150 pA of 500 ms-current pulses were injected with an increment of 10 pA per step, and the firing numbers were quantified for each step.

#### Fiber photometry recording

Calcium-dependent fluorescence signals (470 nm) were background-corrected with autofluorescence signals (405 nm) to control for movement and bleaching artifacts. The laser power was adjusted at the tip of optical fiber to approximately 20–23 µW. Baseline fluorescence (F0) was calculated by the running average of the whole individual recording. All GCaMP6s signal data were normalized to fluorescence change (ΔF/F) by calculating ΔF/F0=(F-F0)/F0. The 15 min timestamps of behavioral event associated with histamine, chloroquine or AEW model-evoked scratching behaviors, air puff was plotted and aligned with fluorescence signal in the custom-written MATLAB codes (R2017b, MathWorks) that were produced by ThinkerTech Nanjing Bioscience. Averaged traces of Ca^2+^ fluorescent signal changes and heatmaps were analyzed using the MATLAB program.

#### Immunohistochemistry

Mice were deeply anesthetized with an 3% isoflurane and perfused with 20 ml ice-cold phosphate-buffered saline (PBS) containing 4% paraformaldehyde (PFA). Brains were carefully removed and postfixed in 4% PFA for 6–8 h, and cryoprotected with 30% sucrose for 48 h. The brain was 30 μm thick sections were coronally prepared using a freezing microtome (Leica CM1950). For immunofluorescent staining, the sections were incubated with PBS containing 0.3% Triton X-100 for 1 h at room temperature and subsequently allowed to react with primary antibodies (rabbit anti-glutamate, 1:500, Sigma; rabbit anti-c-Fos, 1:1000, Synaptic Systems) at 4˚C overnight. After washing with PBS, the sections were subsequently coupled with the corresponding fluorophore-conjugated secondary antibodies for 1.5 h at room temperature. Finally, after rinsing 3 times in PBS, sections were mounted with DAPI staining. Confocal images were acquired under a 10x or 20x objective with a 1024 × 1024 resolution using the Olympus confocal microscopes (FV3000, Olympus). Double fluorescence labelled cells were manually counted using ImageJ software. The Paxinos and Franklin atlas (2013) was employed to define the regions of interest.

#### Itch-induced conditioned place aversion test

To directly test the role of LHb glutamatergic neurons in negative experience of itch, we used itch-related conditioned place aversion (CPA) protocols, based on previous publications [23]. A three-compartment place preference apparatus for mouse was used. One chamber has white walls, the other chamber has black walls and the middle chamber has gray walls as a neutral zone. There were two manual doors between three chambers. The procedure consists of four phases: habituation, pre-test, conditioning and preference testing (post-test). In habituation phase, animals are allowed to freely explore the apparatus to reduce the effects of novelty for 30 min. During pre-test phase, mice were placed into the middle compartment and allowed to freely explore the entire apparatus. We determined the time of one chamber for 15 min as the baseline preference. Following testing of baseline preference, the conditioning phase is performed twice per day (morning and afternoon) for three consecutive days. In the morning, each animal was injected saline (i.d.) into the nape and assigned one compartments for 30 min, which was accordingly designated the “itch-unpaired” compartment. In the afternoon, the same mice received a subcutaneous injection of chloroquine (i.d.) in the opposite compartment (itch-paired compartment) for 30 min. Animals are only allowed to access to one chamber in one conditioning trial. After six conditioning trials, animals are allowed to freely explore the whole apparatus on the preference testing day for 15 min. The time spent in the itch-paired chamber was analyzed.

In the process of the AEW model treatment (0–6 days), we used a chronic itch-CPA test procedure. Mice were placed in a 3-chamber CPA arena and given free access to entire chambers for 30 min on day one. The apparatus was re-designated as a CNO-paired chamber in which the mice were injected with CNO (i.p) and the other as saline-paired chamber. From day 3 to day 5, mice were conditioned for 3 continuous days with two pairing sessions each day. 24 h after the last pairing, mice were given free access to all chambers, and the time spent in the CNO-paired chamber was analyzed.

#### Assessment of motor function

A rotarod system of accelerating treadmills (IITC, Inc., Life Sciences, St. Petersburg, FL) was used to assess coordinate motor activity and general motor disability. On day before the test, mice were placed on an apparatus that accelerated 5–20 revolutions per minute (r.p.m.) for 5 min, and trained to maintain its balancing walking. Mice were gently placed on a rod that gradually accelerated from 5 r.p.m. to 40 r.p.m. over a 5 min period, and the latencies of the mice to drop were recorded.

### Data analysis and statistics

Software used for data analysis and plotting the results included: MATLAB R2017a, Olympus FV10-ASW 4.0a Viewer, GraphPad Prism v.8.0.1, Adobe Illustrator 2021 and Adobe Photoshop 2021. All experiments and data analyses such as immunohistochemistry, electrophysiology and behavior were performed blindly. For the quantitation of c-Fos^+^ cells, we selected 3–4 continuous sections containing LHb from each animal. The average number of c-Fos^+^ neurons in the bilateral LHb per section was calculated as “NO. of c-Fos^+^ neurons”. Statistical detection methods include: student’s paired t test, student’s unpaired t test, Mann-Whitney U test, Wilcoxon signed-rank test, two-way analysis of variance (ANOVA) followed by Bonferroni’s test for multiple comparisons. Before applying the student’s t test statistics, the data were conformed to normal distribution. The data were analyzed with Mann-Whitney U test for unpaired t test and Wilcoxon signed-rank test for paired t test if normality was violated. Statistical significances were represented as *p < 0.05, **p < 0.01, ***p < 0.001, ****p < 0.0001. These data were presented as the mean ± standard error of the mean (SEM).

## Electronic supplementary material

Below is the link to the electronic supplementary material.

Supplementary Material 1

Supplementary Material 2

Supplementary Material 3

## Data Availability

The datasets used and analyzed during the current study are available from the corresponding author on reasonable request.

## Abbreviations

ACC: anterior cingulate cortex
ACSF: artificial cerebrospinal fluid
AEW: Acetone-ether-water model
CPA: conditioned place aversion
CCD: charge-coupled device
CNO: clozapine-N-oxide
EPN: entopeduncular nucleus
GSH: glutathione
Glu^LHb^: glutamatergic neurons within the lateral habenula nucleus
GRPR: gastrin-releasing peptide receptor
HEPES: N-2-hydroxyethylpiperazine-N-2-ethanesulfonic acid
LHb: lateral habenula nucleus
LHA: lateral hypothalamus
mPFC: medial prefrontal cortex
NMDG: Nmethyl-d-glucamine
NAc: nucleus accumbens
PBN: parabrachial nucleus
PAG: periaqueductal gray
PBS: phosphate-buffered saline
PFA: paraformaldehyde
Po: posterior thalamic nucleus
TRPV1: transient receptor potential vanilloid 1
VB: ventral basal nucleus of the thalamus
VTA: ventral tegmental area
