# Supplementary material for: The lateral habenula nucleus regulates pruritic sensation and emotion
Source: Mol Brain. 2023 Jun 27;16:54. doi: 10.1186/s13041-023-01045-7 (PMC10303242; doi:10.1186/s13041-023-01045-7)
Supplement: Supplementary file 1 — Supplementary Material 1 [file 13041_2023_1045_MOESM1_ESM.docx]

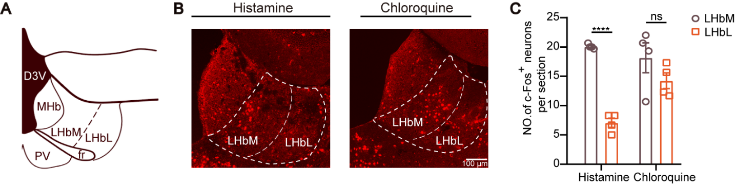
**Fig. S1 c-Fos expression in the medial and lateral LHb from pruritogens-injected mice.**

**A** Diagram of a cross-section about the lateral habenula (LHb). MHb: the medial habenula; LHbM: the medial ‘limbic’ part of the LHb; LHbL: the lateral ‘pallidal’ part of the LHb.

**B** Representative images of c-Fos-positive neurons distribution in the LHb. Scale bar, 100 μm.

**C** Quantification of c-Fos staining in the LHbM and LHbL from pruritogen-treated mice.

Significance was assessed by two-tailed unpaired Student’s *t*-test in (**C**), ****p < 0.0001, not significant (ns). All data were shown as mean ± SEM.
