# Supplementary material for: The lateral habenula nucleus regulates pruritic sensation and emotion
Source: Mol Brain. 2023 Jun 27;16:54. doi: 10.1186/s13041-023-01045-7 (PMC10303242; doi:10.1186/s13041-023-01045-7)
Supplement: Supplementary file 2 — Supplementary Material 2 [file 13041_2023_1045_MOESM2_ESM.docx]

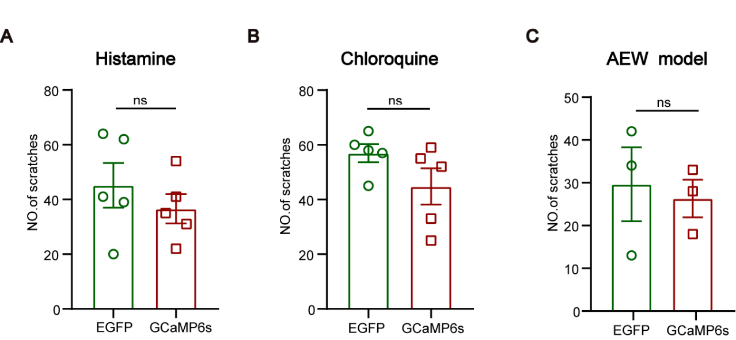
**Fig. S2** **Comparable number of scratches between itch-treated GCaMP6s group and EGFP group.**

**A** The number of scratches in 15 min in acute itch models induced by histamine (related to Fig. 1G).

**B** The number of scratches in 15 min in acute itch model induced by chloroquine (related to Fig. 1H).

**C** The scratching behaviors in 15 min in chronic itch model (related to Fig. 2D).

Significance was assessed by two-tailed unpaired Student’s *t*-test in (**A-C**), not significant (ns). Data were shown as mean ± SEM.
