# Supplementary material for: The lateral habenula nucleus regulates pruritic sensation and emotion
Source: Mol Brain. 2023 Jun 27;16:54. doi: 10.1186/s13041-023-01045-7 (PMC10303242; doi:10.1186/s13041-023-01045-7)
Supplement: Supplementary file 3 — Supplementary Material 3 [file 13041_2023_1045_MOESM3_ESM.docx]

**
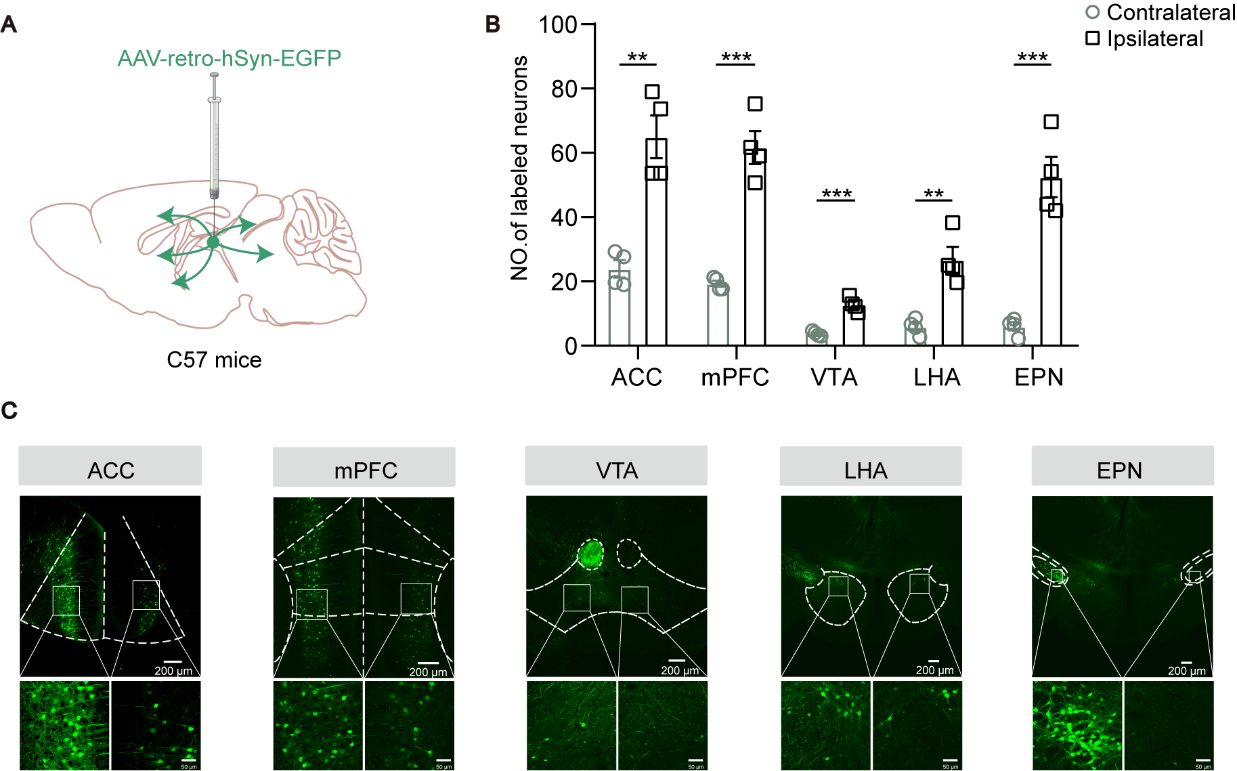
Fig. S3** **LHb upstream brain regions identified by retrograde tracing.**

**A** Schematic of the viral injection.

**B** Comparison of the number of EGFP labeled neurons in the upstream brain regions ipsilaterally or contralaterally to the injection site. (n=3 sections per animal from 4 mice).

**C** Representative images of retrograde EGFP^+^ neurons in the anterior cingulate cortex (ACC), medial prefrontal cortex (mPFC), ventral tegmental area (VTA), entopeduncular nucleus (EPN) and lateral hypothalamus (LHA). Scale bars, 200 μm (left) and 50 μm (right).

Significance was assessed by two-tailed unpaired Student’s *t*-test in (**B**), **p < 0.01, ***p < 0.001. All data were shown as mean ± SEM.
